# Disorder and defects are not intrinsic to boron carbide

**DOI:** 10.1038/srep19330

**Published:** 2016-01-18

**Authors:** Swastik Mondal, Elena Bykova, Somnath Dey, Sk Imran Ali, Natalia Dubrovinskaia, Leonid Dubrovinsky, Gleb Parakhonskiy, Sander van Smaalen

**Affiliations:** 1 Max-Planck-Institut fuer Kohlenforschung, Kaiser-Wilhelm-Platz 1, 45470 Muelheim an der Ruhr, Germany; 2 Laboratory of Crystallography, University of Bayreuth, D-95440 Bayreuth, Germany; 3 Bayerisches Geoinstitut, University of Bayreuth, D-95440 Bayreuth, Germany

## Abstract

A unique combination of useful properties in boron-carbide, such as extreme hardness, excellent fracture toughness, a low density, a high melting point, thermoelectricity, semi-conducting behavior, catalytic activity and a remarkably good chemical stability, makes it an ideal material for a wide range of technological applications. Explaining these properties in terms of chemical bonding has remained a major challenge in boron chemistry. Here we report the synthesis of fully ordered, stoichiometric boron-carbide B_13_C_2_ by high-pressure–high-temperature techniques. Our experimental electron-density study using high-resolution single-crystal synchrotron X-ray diffraction data conclusively demonstrates that disorder and defects are not intrinsic to boron carbide, contrary to what was hitherto supposed. A detailed analysis of the electron density distribution reveals charge transfer between structural units in B_13_C_2_ and a new type of electron-deficient bond with formally unpaired electrons on the C–B–C group in B_13_C_2_. Unprecedented bonding features contribute to the fundamental chemistry and materials science of boron compounds that is of great interest for understanding structure-property relationships and development of novel functional materials.

Boron carbide is one of the hardest substances, surpassed only by diamond and boron nitride^1^. The high mechanical and thermal stability, low density and low costs of fabrication have made boron carbide the prime choice in a series of technological applications^1^,^2^,^3^,^4^,^5^,^6^,^7^. Boron carbide preserves the same structure for a range of compositions, and details of this crystal structure have been discussed in terms of chemical disorder of boron and carbon atoms as well as the presence of vacancies^1^,^8^,^9^,^10^,^11^. Electronic-structure calculations suggest that the properties of boron carbide depend on the stoichiometry and the details of the disorder^2^,^7^,^12^,^13^.

Experimentally, chemical bonding can be accessed through single-crystal x-ray diffraction. Reliable information on the distribution of the electron density in the unit cell can be obtained only for good-quality single crystals with minimal structural disorder^14^. Synthesis of defect-free material is the most challenging task in boron carbide chemistry. We have succeeded in growing small single crystals of the stoichiometric composition B_13_C_2_ by high-pressure–high-temperature techniques (see *Methods*). The material is transparent with a dark red or maroon color, indicating an insulator or a large-band-gap semiconductor. This is in agreement with some literature data^15^, but it is inconsistent with the relatively high electrical conductivity reported for boron carbide^1^. To the best or our knowledge, dark red transparent boron carbide has not been reported before.

A multipole (MP) model has been obtained for the crystal structure of B_13_C_2_ by refinement against accurately measured intensities of Bragg reflections (see *Methods* and Supplementary Information Section S1)^14^. The excellent fit to the diffraction data with R_1_ = 0.0197 provides strong evidence for the stoichiometry of B_13_C_2_, in agreement with the composition obtained by Energy-dispersive x-ray (EDX) analysis (see *Methods*). The excellent fit furthermore indicates the absence of disorder: B_13_C_2_ is composed of B_12_ icosahedral clusters and CBC linear chains (Fig. 1 and Supplementary Information Section S2). Lattice parameters and values of atomic displacement parameters (ADPs) fall within a range previously assigned to the composition B_12_C_3_^1^,^8^,^9^,^10^. The possibility of different compositions was investigated by additional MP refinements with small amounts of carbon at the B_P_ site, corresponding to B_12 + x_C_3 − x_ stoichiometries with x = 0.44 and x = −0.11, respectively (see Supplementary Information Section S1 for details). Both models gave a slightly worse fit to the diffraction data than the B_13_C_2_ model. More importantly, the number of valence electrons of C at the B_P_ site refined to zero, thus showing that the MP refinement has effectively removed carbon from the B_P_ site, providing further support for the ordered stoichiometric character of the investigated crystal. Interestingly, a refinement of the independent atom model (IAM) including the site occupancy factors of C at the B_P_ and B_E_ sites resulted in 19% occupancy of the B_P_ site by carbon (x = −0.11). Contrary to the MP model (R_1_ = 0.0197), the IAM with disorder (R_1_ = 0.0287) leads to only a small improvement of the fit to the data (Table S4). These results suggest that the charge transfer towards B_P_ in the MP model is mimicked in the disordered IAM by a fractional occupancy of the B_P_ site by C.

Discrepancies between the present values of the lattice parameters and ADPs and those reported in the literature^1^,^8^,^9^,^10^ for the same composition may be the result of different degrees of disorder and defects between different samples. The single MP refinement^16^ reported previously for B_13_C_2_ gave a much worse fit to their XRD data (R_1_ = 0.0440), which questions the reliability of that model. The single MP refinement^17^ for B_12_C_3_ also led to a substantially worse fit to their XRD data (R_1_ = 0.0250) than we have obtained for our model against the present XRD data (R_1_ = 0.0197). Thus, a highly precise MP refinement refutes recent less accurate diffraction studies^13^ and theoretical electronic-structure calculations^2^,^12^, where a disorderly replacement by carbon of a certain fraction of the boron atoms of the B_12_ clusters was considered as absolutely essential for the stability of B_13_C_2_.

The MP model extends the independent atom model (IAM) of spherical atoms by parameters describing the reorganization of electron density due to chemical bonding. Previous electron-density studies on boron carbide^18^,^19^ have been restricted to a discussion of the qualitative features of the electron densities. Quantitative information about chemical bonding can be extracted from the static electron density of the MP model through its topological properties according to Bader’s *quantum theory of atoms in molecules* (QTAIM)^14^,^20^. Critical points (CPs) are defined as the positions where the gradient of the electron density is zero [**∇**ρ(**r**) = 0]^20^. They are classified according to the number of positive eigenvalues of the Hessian matrix of second derivatives as local maxima (3 positive eigenvalues), bond critical points BCPs (2), ring critical points RCPs (1) and local minima (0 positive eigenvalues)^20^.

All atomic positions of the present MP model can be identified with local maxima in the static electron density, while additional local maxima do not exist. BCPs and RCPs have been found between the atoms of the B_12_ cluster in a similar pattern as for *α*-boron^21^, and with comparable values for the electron densities and Laplacians (Table 1). Together, these features indicate similar bonding by molecular-type orbitals on the B_12_ clusters in B_13_C_2_ and *α*-boron^21^. According to Wade’s rule^22^, this bonding involves 26 of the 36 valence electrons of the twelve boron atoms of this closo-cluster, thus leaving for each boron atom one orbital but only 5/6 electrons for exo-cluster bonding^21^,^23^.

The crystal structure of B_13_C_2_ comprises four crystallographically independent atoms. CBC chains contain the carbon atom and a boron atom denoted as B_C_; the B_12_ cluster is made of six polar and six equatorial atoms, denoted as B_P_ and B_E_, respectively (Fig. 1). According to the QTAIM^20^, bonding between a pair of atoms exists, if the electron density possesses a BCP between those atoms. For B_13_C_2_, we have found BCPs between pairs of B_P_ atoms from neighboring clusters. The distance B_P_–B_P_ is slightly larger and the magnitudes of the electron density, ρ_BCP_, and Laplacian, ∇^2^ρ_BCP_, are slightly smaller than those of the corresponding inter-cluster bonds in *α*-boron^21^ and γ-boron^24^ (Table 1). The high value of ρ_BCP_ together with a negative value of ∇^2^ρ_BCP_ of large magnitude indicate a strong covalent interaction between these atoms^20^. The similarities with bonding in *α*-boron^21^ (Table 1) allow this bond to be classified as a 2-electron-2-center (2e2c) bond. Further evidence for this interpretation comes from the QTAIM theory, which assigns a charge to each atom by integration of the electron density over the atomic basins. A charge of −0.21 electrons has been obtained by integrating the experimental static electron density over the atomic basin of B_P_ (Table 2). This value is in good agreement with electron counting. With 5/6 electrons per boron atom available for exo-cluster bonding, a formal charge of −0.17 is obtained for B_P_ involved in a 2e2c B_P_–B_P_ exo-cluster bond.

Bond-critical points are also found between a B_E_ atom and the closest C atom. Large magnitudes of ρ_BCP_ and the negative Laplacian ∇^2^ρ_BCP_ indicate a strong covalent interaction and a 2e2c C–B_E_ bond. An equal split of these electrons between C and B_E_ again gives a formal charge of −0.17 for B_E_, and it would result in a (B_12_)^2−^ group^2^ However, carbon is more electronegative than boron and should attract most of the bonding electrons. Indeed, the integration of the electron density over the atomic basins leads to a positive atom B_E_ and a strongly negative C atom (Table 2). A detailed analysis of the electron density shows that the positive charge of B_E_ is the result of a strong polar-covalent character of the C–B_E_ bond, with the BCP much closer to B_E_ than to C (Fig. 2; Table 1), but with a large value of ρ_BCP_ as opposed to an expected small value for ionic bonding^20^.

With the interpretation of B_P_–B_P_ and C–B_E_ bonds as 2e2c bonds, only three electrons are left for the two C–B_C_ bonds of the CBC group (see Supplementary Material Section S3). These bonds can therefore be described as a three-electron-three-center (3e3c) bond or as resonance between two equivalent combinations of one 2e2c and one 1e2c bond (Fig. 3). The large values of the electron density along the bond path (Fig. 2a) correlate with the short bond length, which is explained by the internal pressure on the CBC group^2^. Large magnitudes of ρ_BCP_ and ∇^2^ρ_BCP_ indicate a covalent interaction. The electron deficient character of this bond is in complete agreement with the ionic charge of +2.30 of B_C_. The latter value is the result of the extremely small volume of the atomic basin of this atom, which demonstrates that the internal pressure has squeezed out most of the electrons of B_C_, reminiscent of the effect of pressure on the electrons in lithium metal^25^.

A 3e3c C–B_C_–C bond contains one unpaired electron per formula unit B_13_C_2_. Experimentally, unpaired spins have been observed at much lower concentrations in boron carbides of different compositions^2^,^4^,^5^,^26^,^27^. One explanation lies in chemical disorder and vacancies, which are necessarily present for other compositions than stoichiometric B_13_C_2_, and which reduce the number of unpaired spins. On the other hand, the itinerant character of the electron states or localization as bipolarons may be in agreement with low concentrations of unpaired spins^2^,^5^,^12^. The presence of an unsaturated bond on the CBC chains should result in a high chemical reactivity of this bond. However, we have found that B_C_ is extremely small (Table 2) and well shielded from the outside by C atoms and bulky B_12_ clusters. Steric effects hindering access to reactive sites is known to stabilize radicals^28^,^29^. High temperatures can overcome these barriers, and a high reactivity at elevated temperatures towards oxidizing agents has been described for boron carbide^30^. Recently, amorphisation^6^,^31^ of boron carbide B_12_C_3_ has been explained on the basis of the presence of carbon atoms at a small fraction of the B_P_ sites^32^. Stoichiometric B_13_C_2_ is a form of boron carbide that lacks this detrimental property of technical boron carbide with compositions on the carbon-rich side of B_13_C_2_.

In summary, we have synthesized stoichiometric boron carbide B_13_C_2_, which is free of intrinsic disorder, and is built of B_12_ icosahedral clusters and C–B_C_–C chains. Unlike band-structure calculations^2^,^12^ on fully ordered B_13_C_2_, the ordered stoichiometric compound is an insulator or large band-gap semiconductor. An experimental electron-density study by X-ray diffraction conclusively determines that B_13_C_2_ is an electron-precise material. The electron-deficient character is explained by B_C_ being stripped of two of its valence electrons and the existence of a unique, electron deficient 3e3c bond on the C–B_C_–C chains. The low chemical reactivity follows from the extremely small volume of B_C_.

## Methods summary

### Crystal growth

Single crystals of boron-carbide were grown at high pressures of 8.5–9 GPa and high temperatures of 1873–2073 K using a 1200-ton (Sumitomo) multi-anvil hydraulic press at the Bayerisches Geoinstitut. Energy-dispersive x-ray (EDX) analysis has been employed to determine the composition as B_6.51(12)_C, in agreement with stoichiometric B_13_C_2_. The presence of other elements could be excluded.

### X-ray diffraction experiment for/and electron density analysis

A single crystal of boron-carbide of dimensions 0.09 × 0.08 × 0.05 mm^3^ was chosen for an x-ray diffraction experiment with synchrotron radiation at beamline F1 of Hasylab, DESY in Hamburg, Germany. The sample was kept at a temperature of 100 K, while a complete data set of accurate intensities was measured for Bragg reflections up to sin(θ)/λ = 1.116 Å^−1^. The diffraction data were integrated using the computer program EVAL^33^. Structure refinements have been performed with the software XD2006^34^. A topological analysis of the static electron density has been performed by the modules TOPXD and XDPROP of the computer program XD2006. Two-dimensional density maps have been generated by the module XDGRAPH. See the Supplementary Information for details on procedures and the MP model.

## Additional Information

**How to cite this article**: Mondal, S. *et al*. Disorder and defects are not intrinsic to boron carbide. *Sci. Rep.* **6**, 19330; doi: 10.1038/srep19330 (2016).

## Supplementary Material

Supplementary Information

## Figures and Tables

**Figure 1:**
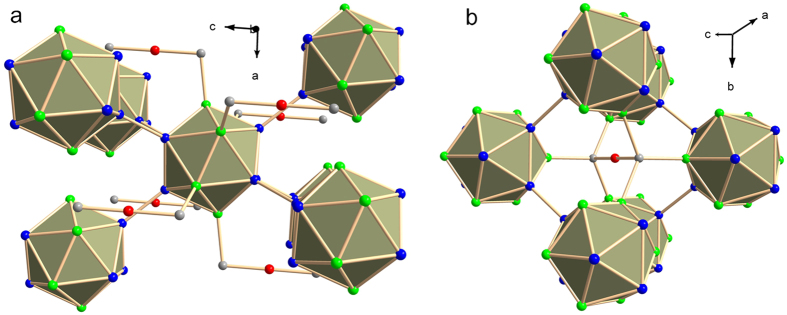
Crystal structure of B_13_C_2_. (**a**) Perspective view highlighting the environment of the icosahedral B_12_ cluster. Each B_12_ cluster is bonded by B_P_–B_P_ bonds to three B_12_ clusters in each of the two neighboring close-packed planes, and to six CBC chains by C–B_E_ bonds. (**b**) Perspective view highlighting the environment of the CBC chain. Each carbon atom is bonded to three B_12_ clusters within a single close-packed plane. Color code: B_P_ is blue, B_E_ is green, B_C_ is red, and C is grey.

**Figure 2:**
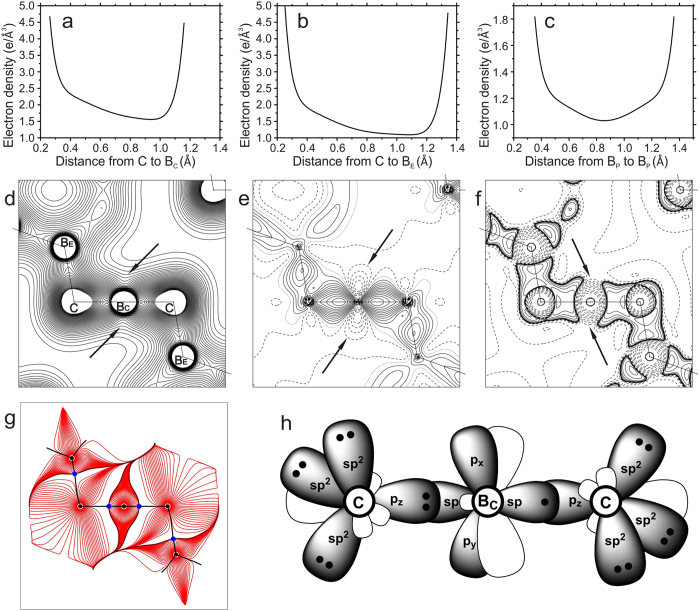
ED distribution in *exo*-cluster bonds and orbital hybridization scheme. (**a**) Electron density along the C–B_C_ bond path. (**b**) ED along the C–B_E_ bond path. (**c**) ED along the inter-cluster B_P_–B_P_ bond path. (**d**) ED distribution in the B_E_–C–B_C_ plane. Contour lines are at 0.05 *e*Å^−3^ up to 2.00 *e*Å^−3^. A groove-like feature in the ED around the B_C_ atom (indicated by arrows) suggests the absence of electrons. (**e**) Dynamic deformation density map^18^ in the same plane. Contour lines are at 0.05 *e*Å^−3^ intervals; positive, negative and zero contours are drawn as solid, dashed and dotted lines respectively. Negative ED contours in the region shown by the arrows indicate empty 2*p* orbitals of B_C_. (**f**) Laplacian in the same plane showing the valence shell charge concentrations (VSCC) around each atom. Contour lines at ±(2, 4, 8) × 10^*n*^ *e*Å^−5^ (−3 ≤ *n* ≤ 3). No VSCC has been found in the regions indicated by arrows, again pointing to empty 2*p* orbitals of B_C_. (**g**) Gradient trajectories of the ED with BCPs (blue dots) indicated. It can be noticed that the volume of the B_C_ atomic basin is small and trajectories inside the basin are squeezed along the direction perpendicular to CBC chain indicating depletion of electrons. (**h**) The orbital hybridization scheme for C and B_C_ atoms. Filled orbitals are indicated by black dots representing electrons. The orbitals *p*_*x*_ and *p*_*y*_ of the atom B_C_ are empty.

**Figure 3:**
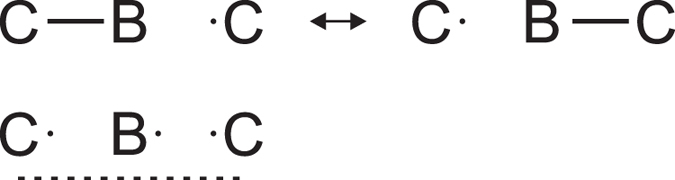
Resonance representation of the 3e3c bond on CBC chains.

**Table 1:** Geometries and topological properties of the experimental static electron density for 2-center *exo*-cluster bonds in B_13_C_2_.

Bond	*d* (Å)	*d*_BCP_ (Å)	*ρ*_BCP_ (*e*/Å^3^)	∇^2^*ρ*_BCP_(*e*/Å^5^)
**Bonds in B_13_C_2_**				
**C**–**B**_**E**_	1.6037(2)	1.082/0.523	1.097	−8.289
**C**–**B**_**C**_	1.4324(5)	0.938/0.494	1.556	−8.985
**B**_**P**_–**B**_**P**_	1.7131(4)	0.857/0.857	1.030	−6.463
**Inter-cluster bonds in α-boron**				
**B1**–**B1** (*2e2c*)^21^	1.6734(3)	0.837/0.837	1.104	−9.572
**Inter-cluster bonds in γ-boron**				
**B3**–**B3** (*2e2c*)^24^	1.6599(5)	0.830/0.830	1.165	−10.404
**B1**–**B4** (*1e2c*)^24^	1.8275(2)	0.865/0.979	0.782	−4.002

*d* is the bond-length and *d*_BCP_ is the distance between a BCP and each of the two constituent atoms of that bond. *ρ*_BCP_ is the electron density at the BCP and ∇^2^*ρ*_BCP_ is its Laplacian. Topological properties for the inter-cluster B–B bonds in α-boron^21^ and in γ-boron^24^ are also given.

**Table 2:** Atomic basins (volume V_Basin_) and ionic charges for the four crystallographically independent atoms in B_13_C_2_ along with their multiplicity in the unit cell.

Atom	Multiplicity	V_Basin_ (Å^3^)	Charge (*e*)
**B**_**P**_	6	7.808	−0.210
**B**_**E**_	6	5.176	+ 0.703
**C**	2	14.571	−2.610
**B**_**C**_	1	1.936	+ 2.298
