# Supplementary material for: Disorder and defects are not intrinsic to boron carbide
Source: Sci Rep. 2016 Jan 18;6:19330. doi: 10.1038/srep19330 (PMC4725998; doi:10.1038/srep19330)
Supplement: Supplementary Information [file srep19330-s1.pdf]

Supplementary information:

Disorder and defects are not intrinsic to boron carbide.

Swastik Mondal[1,2]*, Elena Bykova[2,3], Somnath Dey[2], Sk Imran Ali[2], Natalia Dubrovinskaia[2], Leonid Dubrovinsky[3], Gleb Parakhonskiy[2,3], Sander van Smaalen[2]*

[1]Max-Planck-Institut fuer Kohlenforschung, Kaiser-Wilhelm-Platz 1, 45470 Muelheim an der Ruhr, Germany

[2]Laboratory of Crystallography, University of Bayreuth, 95440 Bayreuth, Germany

[3]Bayerisches Geoinstitut, University of Bayreuth, 95440 Bayreuth, Germany

*Email: mondal@mpi-muelheim.mpg.de; smash@uni-bayreuth.de

Section S1. The multipole model

X-ray diffraction experiments have been performed at beamline F1 of Hasylab, DESY, Hamburg, Germany. Data have been analysed and processed as described in the 'Methods' section. Table S1 summarizes the crystallographic and experimental parameters. Refinements of the multipole (MP) model[14] have been performed against the diffraction data, employing reflections with intensities larger than three times their standard uncertainties. They revealed a structure model comprising three B_{12} clusters and three CBC linear chains in the unit cell. Atomic coordinates and atomic displacement parameters from the MP refinement are listed in Table S2. Table S3 contains the values of all multipole parameters.

Lattice parameters of our sample (Table S1) as well as anisotropic displacement parameters (ADPs) of the B_C atom (Table S2) possess values within the range of values in the literature assigned to compositions close to $B_{12}C_3$[1,8-10]. In order to investigate the possibility of different compositions, we have performed additional refinements. Initially, the independent atom model (IAM) was refined with SHELX[35] for various compositions (Table S4). The carbon atom C2 was placed at the B_P site with positional and ADP parameters of B_P and C2 constrained to be equal. The site occupation factor (s.o.f.) of C2 was freely refined, while s.o.f.[B_P] + s.o.f.[C2] was constrained to be 1. The refinement converged at 0.19 occupancy of the B_P site by carbon, within one standard uncertainty of 0.11 in x equal to the composition $B_{12}C_3$ presumed on the basis of the literature[1,8-10] on lattice parameters and ADPs. However, the fit to the XRD data is only slightly better than that of the $B_{13}C_2$ composition (Table S3) and this small improvement cannot compete with the much better fit to the XRD data of the MP model as compared to the IAM model. In a second disorder model, carbon was allowed at both the B_P and B_E positions, leading to a similar occupancy of the B_P site by carbon as before and only a small amount of carbon at the B_E site, while the fit to the XRD data is virtually the same as the B_P/C2 disorder model (Table S4). Refinements failed for an independent variation of the atomic parameters of C2 and B_P, due to the high correlations between parameters.

Based on the results with the IAM (Table S4) we have performed additional refinements of the MP model. XD2006 does not allow refinement of the s.o.f.. Therefore, we have performed refinements with s.o.f.[C2] fixed to 0.186, corresponding to the refined composition of the IAM. A third MP refinement was performed with s.o.f.[C2] = 0.093. In both cases, the monopole parameter M1[C2] refined to a small negative value that can be considered to be zero within standard uncertainties. This implies that the MP refinement removes all valence electrons of carbon from the model. The number of core electrons is not varied in the MP refinements. Taking into account the fractional occupancies of the B_P/C2 site, the number of core electrons add up to exactly 2, as they are required for a boron atom.

The IAM refinements places a fraction of 0.186 of carbon at the B_P site (Table S4); the MP refinement then removes any carbon from the B_P site (Table S5). One can thus conclude that the deviation from the IAM of the electron density is mimicked in the IAM refinement by an apparent partial occupation of the B_P site by carbon. The much lower R value for the MP model than for the IAM demonstrates that our sample does not involve any disorder of the boron and carbon atoms.

The static electron density corresponding to the MP model has been generated and analyzed with the XD2006 suite of software[34]. Topological properties of this electron density are given in Table S6 and a summary of these data is given in Table 1.

Table S1. Crystallographic data for boron-carbide. Obverse setting on a hexagonal unit cell.

Chemical formula	$B_{13}C_2$
Temperature (K)	100
Crystal system	Trigonal
Space group	*R-3m*
Z	3
a (Å)	5.5962 (3)
c (Å)	12.0661 (7)
Volume(Å^3)	327.25 (3)
R centering (obverse setting)	(2/3, 1/3, 1/3); (1/3, 2/3, 2/3)
Diffraction experiment	
crystal dimension (mm^3)	0.09 × 0.08 × 0.05
Radiation type	synchrotron
Wavelength, λ (Å)	0.5600
$[\sin(\theta)/\lambda]_{max}$ (Å^{-1})	1.116
Observed criterion	$I > 3\sigma_I$
No. of observed/all reflections	440/505
Multipole refinement:	
R_F(obs)	0.0197
wR_F^2	0.0227
wR_F^2(obs)	0.0290
GoF(obs)	1.7435
Nref/Nv	7.5862
$\Delta\rho_{max}$ /$\Delta\rho_{min}$ (eÅ^{-3})	0.248/-0.235

Table S2. Relative coordinates and atomic displacement parameters (Å^2) of the four crystallographically independent atoms in the final multipole model of $B_{13}C_2$.

Atom	C	B_E	B_P	B_C
x	0	0.16299(3)	0.22580(3)	0
y	0	-0.16299(3)	-0.22580(3)	0
z	0.61871(4)	0.64153(2)	0.78058(3)	0.5
U_{11}	0.00429(9)	0.00417(7)	0.00389(7)	0.00809(16)
U_{22}	0.00429(9)	0.00417(7)	0.00389(7)	0.00809(16)
U_{33}	0.00515(16)	0.00607(10)	0.00583(11)	0.0060(3)
U_{12}	0.00215(4)	0.00217(7)	0.00183(8)	0.00404(8)
U_{13}	0	-0.00004(4)	-0.00006(4)	0
U_{23}	0	0.00004(4)	0.00006(4)	0

Table S3. Multipole parameters of the four crystallographically independent atoms in the final multipole model of $B_{13}C_2$.

**Parameter**	**Value**	**s.u.**
**Atom C**		
M1	0.586326	0.012493
D0	-0.000168	0.004935
Q0	0.016447	0.003971
O0	0.022055	0.004522
O3+	0.015597	0.003634
H0	-0.002325	0.005362
H3+	-0.005897	0.004569
κ	1.012226	--
κ'	0.966884	--
**Atom B_E:**		
M1	1.571910	0.019892
D1+	0.042662	0.028498
D1-	-0.019837	0.018612
Q0	-0.011424	0.010417
Q2+	-0.001063	0.028631
Q2-	0.053554	0.016523
O1+	0.062015	0.013601
O1-	0.074578	0.014509
O3+	0.119004	0.009174
O3-	-0.064152	0.036925
H0	0.018670	0.041184
H2+	0.003407	0.023223
H2-	-0.010762	0.032825
H4+	0.006101	0.035684
H4-	-0.037513	0.026519
κ	0.972780	--
κ'	0.964800	--

**Atom B_P:**		
M1	1.505679	0.015983
D1+	0.010013	0.011298
D1-	-0.004375	0.009411
Q0	-0.030558	0.021826
Q2+	-0.040192	0.009815
Q2-	0.055342	0.012067
O1+	0.058693	0.027575
O1-	0.053614	0.021749
O3+	0.067507	0.016117
O3-	-0.037684	0.012449
H0	0.000336	0.023600
H2+	0.005435	0.029034
H2-	0.017810	0.026510
H4+	-0.001817	0.021047
H4-	0.004564	0.019062
κ	1.055122	--
κ'	0.991027	--
**Atom B_C:**		
M1	0.252684	0.011119
Q0	0.046158	0.004713
H0	0.003399	0.006817
H3+	-0.007520	0.005064
κ	0.975455	--
κ'	0.987664	--

Table S4. Refinements of the IAM for $B_{12+x}C_{3-x}$ for various compositions x. Experimental data, lattice parameters and symmetry are given in Table S1. Compositions x have been calculated from the refined site occupation factors (s.o.f.) according to x = 1 – 6×s.o.f.[C2] – 6×s.o.f.[C1]. A negative value of x means more than 3 C atoms per formula unit. Constraints are s.o.f.[B_P] = 1-s.o.f.[C2] and s.o.f.[B_E] = 1-s.o.f.[C1].

Chemical formula	$B_{13}C_2$	$B_{12+x}C_{3-x}$	$B_{12+x}C_{3-x}$
Composition x	1	-0.11 (11)	-0.50 (17)
at.-% Carbon	13.3	20.8	23.3
R_F(obs)	0.0295	0.0287	0.0286
wR_F^2	0.0876	0.0808	0.0801
wR_F^2(obs)	0.0837	0.0769	0.0763
GoF(obs)	1.176	1.153	1.156
No. of parameters	18	19	20
$\Delta\rho_{max}$ (eÅ^{-3})	0.747	0.727	0.734
$\Delta\rho_{min}$ (eÅ^{-3})	-0.286	-0.284	-0.282
s.o.f.[B_P]	1	0.814 (19)	0.79 (2)
s.o.f.[C2]	0	0.186 (19)	0.21 (2)
s.o.f.[B_E]	1	1	0.96 (2)
s.o.f.[C1]	0	0	0.04 (2)
U11[B_C] (Å^2)	0.0075	0.0074	0.0074
U33[B_C] (Å^2)	0.0068	0.0066	0.0066

Table S5. Refinements of the MP model of $B_{12+x}C_{3-x}$ for three compositions x. Experimental data, lattice parameters and symmetry are given in Table S1. Values for $B_{13}C_2$ have been taken from Tables S1, S2 and S3.

Chemical formula	$B_{13}C_2$	$B_{12+x}C_{3-x}$	$B_{12+x}C_{3-x}$
Composition x	1	0.44	-0.1148
at.-% Carbon	13.3	17.1	20.8
R_F(obs)	0.0197	0.0210	0.0209
wR_F^2(obs)	0.0290	0.0316	0.0312
GoF(obs)	1.744	1.941	1.921
No. of parameters	58	73	73
$\Delta\rho_{max}$ (eÅ^{-3})	0.248	0.337	0.338
$\Delta\rho_{min}$ (eÅ^{-3})	-0.235	-0.290	-0.285
s.o.f.[B_P]	1	0.907	0.8142
s.o.f.[C2]	0	0.093	0.1858
M1[B_P]	1.51 (2)	2.25 (8)	2.26 (8)
No. electrons [B_P]	6.02 (4)	6.5 (2)	6.5 (2)
M1[C2]	-	-0.011 (57)	-0.015 (56)
No. electrons [C2]	-	1.98 (11)	-1.97 (11)
U11[B_C] (Å^2)	0.0081	0.0074	0.0081
U33[B_C] (Å^2)	0.0060	0.0066	0.0057

Table S6. Geometries and topological properties of the experimental static electron density for *intra-* and *exo*-cluster bonds in $B_{13}C_2$. d is the bond-length and d_{BCP} is the distance between a BCP and each of the two constituent atoms of that bond. ρ_{BCP} is the electron density at the BCP and $\nabla^2\rho_{BCP}$ is its Laplacian. Topological properties are also included for the inter-cluster B–B bonds in α-boron[21] and in γ-boron[24].

**Bond**	***d*** **(Å)**	d_{BCP} **(Å)**	ρ_{BCP} **(*e*/Å^3)**	$\nabla^2\rho_{BCP}$ **(*e*/Å^5)**
**Intra-cluster bonds in $B_{13}C_2$**				
**B_P–B_P**	1.8053(4)	0.878/0.932	0.736	-0.556
**^1B_P–B_E**	1.7997(4)	0.972/0.834	0.761	-1.942
**^2B_P–B_E**	1.7848(5)	0.912/0.874	0.802	-1.675
**B_E–B_E**	1.7590(3)	0.886/0.886	0.742	-1.991
**Bonds involving CBC in $B_{13}C_2$**				
**C–B_E**	1.6037(2)	1.082/0.523	1.097	-8.289
**C–B_C**	1.4324(5)	0.938/0.494	1.556	-8.985
**Inter-cluster bonds in $B_{13}C_2$**				
**B_P–B_P**	1.7131(4)	0.857/0.857	1.030	-6.463
**Inter-cluster bonds in α-boron**[21]				
**B1–B1** (*2e2c*)[21]	1.6734(3)	0.837/0.837	1.104	-9.572
**Inter-cluster bonds in γ-boron**[24]				
**B3–B3** (*2e2c*)[24]	1.6599(5)	0.830/0.830	1.165	-10.404
**B1–B4** (*1e2c*)[24]	1.8275(2)	0.865/0.979	0.782	-4.002

Section S2. Crystal structure of $B_{13}C_2$ stoichiometric boron carbide.

The crystal structure of $B_{13}C_2$ comprises two structural units: a linear chain CBC and a B_{12} cluster with distorted icosahedral structure—ubiquitous in boron and boron-rich compounds. The crystal structures of $B_{13}C_2$, α-boron[21] and γ-boron[24] are based on a cubic closest packing of spheres (ccp), with B_{12} clusters assuming the role of "spheres." In α-boron[21], the ccp

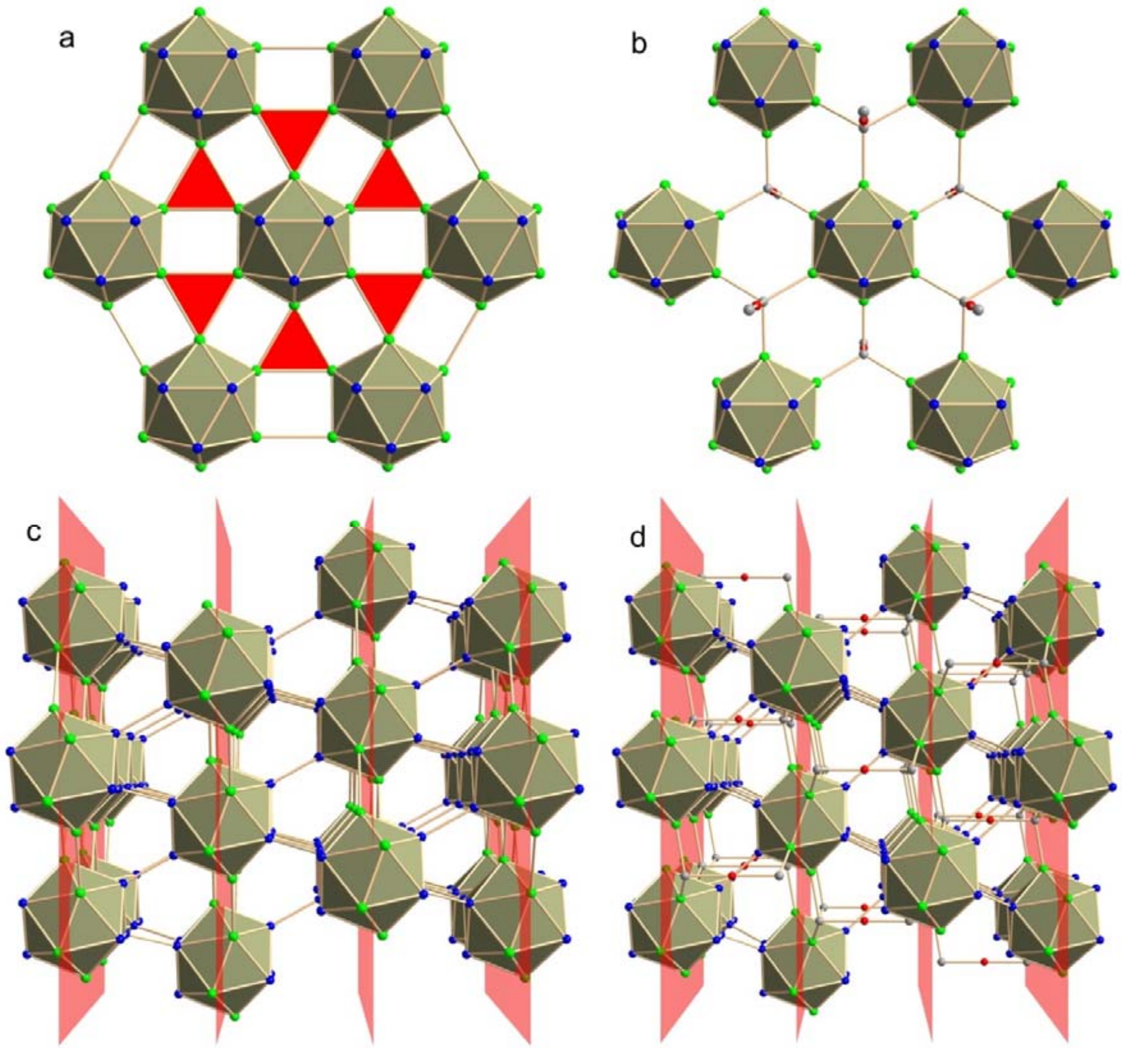

Figure S1 | Crystal structures of $B_{13}C_2$ boron-carbide and α-boron.
a. Crystal structure of α-boron viewed along the c-axis. *2e3c* inter-cluster bonds are shown as red triangles. **b.** Perspective view along the c-axis of the crystal structure of $B_{13}C_2$. Neighboring clusters are connected via CBC chains. **c.** Perspective view along the a-axis of the crystal structure of α-boron. Close-packed layers perpendicular to the c-axis are highlighted by transparent red planes. **d.** Perspective view along the a-axis of the crystal structure of $B_{13}C_2$. CBC chains are visible at octahedral sites between the close-packed layers of B_{12} clusters. B_E, B_P and B_C atoms are drawn in the colors green, blue and red, respectively; C atoms are gray.

structure is rhombohedrally distorted; each B_{12} cluster is bonded to its six neighbors within the close-packed plane by *2e3c* bonds (Fig. S1**a**), and it is bonded to the other six neighbors

by inter-cluster, *2e2c* B_P–B_P bonds (Fig. S1**c**). $B_{13}C_2$ is rhombohedrally distorted as well, but with the octahedral voids occupied by linear CBC groups located on the threefold rotoinversion axis; the *2e3c* bonds of α-boron are replaced by three C–B_E bonds (Fig. S1**b**), while the second C atom of CBC makes an equivalent set of bonds within a neighboring layer (Fig. S1**d**). γ-boron is orthorhombically distorted and contains B_2 groups (dumbbells) at the octahedral sites of the ccp structure; here the distortion is responsible for a reduction of the connectivity of B_{12} clusters[24].

Thus, the crystal structure of $B_{13}C_2$ can be considered as stuffed version of the α-boron structure with the CBC chains squeezed between layers of B_{12} clusters.

The CBC linear chain is a structural unit in several other compounds, especially metal-boron carbides (Table S7). It appears that bond lengths C–B_C possess comparable values in different compounds, with the bond length in $B_{13}C_2$ being the shortest among all compounds.

Table S7: Bond lengths C–B_C (Å) in linear CBC units in various chemical compounds.

**No.**	**bond-length C–B_C (Å)**	**Compound**	**Lattice system, space group**	**Reference**
1.	1.446(7)	Lu_3BC_3	Orthorhombic, *Cmcm*	[37]
2.	1.47(6)	$Ce_5B_2C_6$	Tetragonal, *P4*	[38]
3i.	1.484	Sc_2BC_2	Tetragonal, *I4/mmm*	[39]
3ii.	1.4747(1)	Sc_2BC_2	Tetragonal, *I4/mmm*	[40]
4.	1.441	Al_3BC_3	Hexagonal, *P-3c1*	[35]
5.	1.48(2)	$Gd_4B_3C_4$	Triclinic, *P-1*	[41]
6.	1.4460(7)	$Mg_3B_{50}C_8$	Monoclinic, *C2/m*	[42]
7.	1.4324(5)	$B_{13}C_2$	Rhombohedral, *R-3m*	[Present work]

Section S3. Chemical bonding in stoichiometric $B_{13}C_2$ boron carbide.

According to Wade's rule, the B_{12} closo-cluster uses 26 of its 36 valence electrons for molecular-orbital-type bonding on the clusters, leaving for exo-cluster bonding 12 sp hybrid orbitals perpendicular to the surface of the clusters, but only 10 electrons. We have recently demonstrated the validity of this model for B_{12} within both α-boron[21] and γ-boron[24]. The present experimental results indicate it to be valid for $B_{13}C_2$ as well. A simplified model, assigning 2e2c character to all exo-cluster bonds, B_P–B_P, C–B_E and C–B_C, then results in a singly charged compound $(B_{13}C_2)^-$ [*Ref.* 2], thus illustrating the electron-deficient character of $B_{13}C_2$. In the literature it has been suggested that replacement of one boron atom with carbon results in an electron precise compound $B_{12}C_3$ (B_4C), but its structure is disordered, with one of the carbon atoms randomly replacing one of the B_P atoms in each B_{12} cluster[2,12,13].

The topological analysis (Table 1) of the experimental static electron density of $B_{13}C_2$ reveals bond critical points (BCPs) for each pair of atoms B_P–B_P, C–B_E and C–B_C, indicating bonding interactions between those atoms. The bond-length B_P–B_P, is slightly longer than the length of

the corresponding bond in α-boron[21], reflecting the negative internal pressure exerted by the CBC groups upon the inter-cluster bonds. Together with an electron density ρ_{BCP} that is marginally lower than in α-boron, this provides evidence for a *2e2c* character of the exo-cluster B_P–B_P bond. The bond length and electron density at the BCP indicate a *2e2c* character for the C–B_E bond too. Related to its very short distance, the C–B_C bond has a high value of ρ_{BCP}. Nevertheless, the electron count leaves only three electrons for the two C–B_C bonds of a single CBC group. We interpret these bonds as a *3e3c* bond, which might be seen as resonance between the two equivalent configurations of a *2e2c* and a *1e2c* bond [Fig. 3]. The uncompensated spin should be responsible for weak paramagnetism of boron carbide[4,26], which might be removed due to the itinerant character of the electrons. Shielding by the bulky B_{12} groups may explain the chemically inert property of the formally radical group CBC.

This interpretation of chemical bonding in $B_{13}C_2$ is in complete agreement with the integrated properties of the experimental electron density. Neutral atoms possess 5/6 electrons for exo-cluster bonding of each atom of B_{12}, three valence electrons for B_C and four valence electrons for C. The topological analysis results in a charge of -0.21 for B_P, in reasonable agreement with a formal charge of -1/6 in case of a 2e2c exo-cluster B_P–B_P bond. Despite the fact that B_E contributes 5/6 and C contributes 7/6 electrons to the 2e2c C–B_E bond, B_E is positively charged (+0.70) and carbon is a negative ion with charge -2.61. This can be explained by the polar-covalent character of this bond, as revealed by the asymmetric location of the BCP (distance 1.082 Å from C and 0.523 Å from B_E). The highly negative charge of C is furthermore explained by the unique topology of the atomic basin of B_C: The volume of B_C is extremely small (Table 2), while the shape of the atomic basin demonstrates that the space where the p_x and p_y orbitals would be located actually belongs to the basins of the carbon atoms. Accordingly B_C is highly positive with a charge of +2.30. These features are in agreement with a model where two valence electrons of B_C are squeezed out by internal pressure, much as Li can lose an electron by external pressure[21].

Additional References:

35. Sheldrick, G. M. A short history of SHELX. *Acta Crystallogr. A* **64**, 112–122 (2008).
36. Hillebrecht, H. & Meyer, F. D. Synthesis, structure, and vibrational spectra of Al_3BC_3, a carbidecarboborate of aluminum with linear $(C{=}B{=}C)^{5-}$ anions. *Angew. Chem. Int. Ed.* **35,** 2499-2500 (1996).
37. Oeckler, O. *et al.* Synthesis, characterization, structural and theoretical analysis of a new rare-earth boride carbide: Lu_3BC_3. *Z. Anorg. Allg. Chem.* **627**, 1389-1394 (2001).
38. Bauer, J. & Bars, O. The crystal structure of the carbon-rich rare earth borocarbide $Ce_5B_2C_6$. *J. Less-Common Met.* **83,** 17-27 (1982).
39. Halet, J. -F., Saillard, J.-Y. & Bauer, J. Electronic structure of the new rare earth borocarbide Sc_2BC_2. *J. Less-Common Met.* **158,** 239-250 (1990).

40. Shi, Y., Leithe-Jasper, A. & Tanaka, T. New ternary compounds $Sc_3B_{0.75}C_3$, $Sc_2B_{1.1}C_{3.2}$, $ScB_{15}C_{1.60}$ and subsolidus phase relations in the Sc–B–C system at 1700°C. *J. Sol. State Chem.* **148,** 250-259 (1999).
41. Jardin, C. *et al.* Synthesis, characterization, and structural and theoretical analysis of $Gd_4B_3C_4$: a novel rare earth metal borocarbide containing two different boron-carbon arrangements. *Inorg. Chem.* **39,** 5895-5900 (2000).
42. Adasch, V. *et al.* Synthesis, Crystal Structure, and Properties of $Mg_xB_{50}C_8$ or $Mg_x(B_{12})_4(CBC)_2(C_2)_2$ (x = 2.4-4). *J. Am. Chem. Soc.* **132,** 13723-13732 (2010).
